# *APOE4* to *APOE2* ‘Switching’ in the CNS vs Periphery: Differential Effects on AD Neuropathology

**DOI:** 10.1002/alz70855_100273

**Published:** 2025-12-23

**Authors:** Lance A Johnson

**Affiliations:** ^1^ University of Kentucky, Lexington, KY, USA

## Abstract

**Background:**

Compared to individuals carrying two copies of the E4 allele of Apolipoprotein E (APOE), E2 homozygotes have a ∼99% reduction in late‐onset Alzheimer's disease (AD) risk. Given its strong risk profile and multitude of biological effects, APOE presents as a clear candidate for gene therapy. In principle, a full 'correction' of E4 to the highly neuroprotective E2 allele could delay or prevent AD for millions of individuals. Here, we aimed to determine the therapeutic potential of cell‐type and pool‐specific (CNS vs periphery) *APOE* allele replacement and explore potential mechanisms therein.

**Method:**

We assessed the physiological and neuropathological changes associated with an *in vivo APOE4* to *APOE2* transition selectively in hepatocytes, astrocytes or microglia using a novel transgenic *APOE* “switch mouse” (APOE4s2) model. These mice express a floxed human *APOE4* coding region followed by the human *APOE2* coding region. mRNA and proteomic analyses were employed to validate an efficient E4 to E2 transition in target cell‐types. Behavioral measures and a suite of neuropathological analyses were applied to assess the effects of an late‐stage allelic switch on amyloid, cognition, and plaque‐associated glial reactivity.

**Result:**

RNA and protein analyses confirm that APOE4s2 mice synthesize *APOE4* pre‐switch, and that tamoxifen induces efficient recombination and expression of human *APOE2* in target tissues. Single‐cell transcriptomics reveals that glia‐selective replacement of *APOE4* with *APOE2* results in distinct cell‐autonomous and non‐autonomous alterations to the transcriptome. Neuropathological analyses show that while both an astrocyte‐ and microglia‐specific E4 to E2 ‘switch’ significantly decrease total amyloid burden, the effects of a hepatocyte‐specfic APOE replacement are much more subtle. Further, there are unique (and sometimes disparate) cell‐type driven responses in gliosis and cognitive outcomes.

**Conclusion:**

Together, these data suggest that while either an astrocyte‐ or microglia‐specific E4 to E2 replacement is sufficient to mitiage amyloid pathology, the cellular response to AD pathology differs substantially depending on the cell type targeted. Overall, this proof of concept data provides insight that will be valuable for guiding development of future *APOE* directed therapies.

